# Asymmetric Spatial Processing Under Cognitive Load

**DOI:** 10.3389/fpsyg.2018.00583

**Published:** 2018-04-23

**Authors:** Lien Naert, Mario Bonato, Wim Fias

**Affiliations:** ^1^Department of Experimental Psychology, Ghent University, Ghent, Belgium; ^2^Department of General Psychology, University of Padova, Padua, Italy

**Keywords:** spatial attention, verbal working memory, cognitive load, detection task, visuo-spatial processing

## Abstract

Spatial attention allows us to selectively process information within a certain location in space. Despite the vast literature on spatial attention, the effect of cognitive load on spatial processing is still not fully understood. In this study we added cognitive load to a spatial processing task, so as to see whether it would differentially impact upon the processing of visual information in the left versus the right hemispace. The main paradigm consisted of a detection task that was performed during the maintenance interval of a verbal working memory task. We found that increasing cognitive working memory load had a more negative impact on detecting targets presented on the left side compared to those on the right side. The strength of the load effect correlated with the strength of the interaction on an individual level. The implications of an asymmetric attentional bias with a relative disadvantage for the left (vs the right) hemispace under high verbal working memory (WM) load are discussed.

## Introduction

We are constantly confronted with an amount of information that dramatically exceeds our ability to process it. Our capacity to attentively process information coming from the outside is limited and thus attentional selection is essential. Different types of selective attention can be distinguished (Carrasco, 2011). One type is feature-based attention, where attention is allocated to a specific aspect of objects (e.g., color). Depending on the task, a particular feature is made relevant and often the aim is to focus only on the relevant information and ignore the irrelevant. This is typically investigated in interference paradigms by testing the effect induced by a distractor or a task-irrelevant stimulus. In a Stroop task for instance, where the aim is to name the color of a word, the meaning of the word itself, even though irrelevant, interferes when performing the task (Stroop, 1935). Another type of selective attention is spatial attention, in which the available attentional resources are distributed across space as a function of task demands. Depending on the specific task to be performed, the most fruitful behavior could be to focus on a specific region or to distribute attentional resources across larger areas. A prototypical task used to investigate spatial attention is the Posner cueing paradigm (Posner, 1980). In this paradigm, a lateralized target is preceded by a cue which directs attention toward a specific location in space. In a valid trial, the cue correctly predicts target position while in an invalid trial, the cue predicts a position different from where the target will appear.

An important question is how the mechanisms of selective visual attention operate in dual task situations; that is, in situations in which central processing resources cannot be dedicated to one task only. For instance, consider what happens in traffic. While driving a car you have to select visual information that is relevant (like pedestrians, other cars, traffic signs, etc.) while ignoring irrelevant information (like scenery, houses, etc.). If you receive a phone call while driving, processing resources will have to be divided between driving and the verbal interaction. The question then is to what extent selectively attending to relevant visual information and ignoring irrelevant information is equally efficient as it is without being engaged in a phone call.

This has mainly been addressed by interference paradigms. The load theory by Lavie (1995) looked at how attentional selection is influenced by the level and type of load. Lavie made the important distinction between the effects of perceptual load and cognitive load for determining whether or not peripheral distracters were interfering with performance. Perceptual load is defined as the number and complexity of perceptual operations that the task involves. Cognitive load is described as the total amount of demand imposed on working memory (WM). Lavie and colleagues found that during tasks with a low perceptual load, despite the fact that we try to ignore them, distractors are still processed (Lavie, 1995; de Fockert et al., 2001; Lavie, 2010) whereas when the perceptual load is high, interference from irrelevant information disappears. In another study, the effects of perceptual load were contrasted with cognitive load conditions (Lavie and De Fockert, 2005). Interestingly, the two different types of load had an opposite effect on processing distractors. Whereas high perceptual load improved the ability to focus attention to the relevant and ignore the irrelevant, this process deteriorated under conditions of high cognitive load. Irrelevant distractors were difficult to ignore under high cognitive load and therefore had a strong impact on processing the relevant target information compared to when the target and distractor were presented under conditions of low cognitive load. The different effect observed for perceptual versus cognitive load shows that increasing the load does not always imply an automatic impairing of selective attention processing by disrupting general cognitive control. Kim et al. (2005) propose that the effects of load could depend on whether there is overlap in content-specific processing, thus leading to interference between WM and the selective attention task (but see Gil-Gómez de Liaño et al., 2016).

In this respect it should be made explicit that WM is not a unitary mechanism, but is suggested to have two domain-specific slave systems: the visuospatial sketchpad and the phonological loop (Baddeley and Hitch, 1974; Baddeley, 2003). Traditional models of dual task interference state that the visuospatial and phonological WM resources are to a great extent independent of each other. Consequently, the maintenance of information in verbal WM is not supposed to conflict much with another concurrent task unless it requires verbal processing too. A study by Marciano and Yeshurun (2017) revealed a recurrent difficulty for replicating the load theory’s predictions concerning the perceptual load and distractibility. They found substantial inter-individual differences about the impact load had. Frequently, the results showed an opposite pattern to what was expected based on the load theory. Under low levels of perceptual load, participants with a low WM capacity show greater distractor processing than those with a high WM capacity (Shipstead et al., 2012), showing that WM can influence the visual distractibility. Marciano and Yeshurun (2017) investigated if their results could be explained by inter-individual differences, by testing the same participants several times. The results showed large between-sessions variations, indicating that not only inter-, but also intra-individual differences should be taken into account to have a better understanding of what is actually being measured.

As above reviewed, the load theory of Lavie (1995) focuses on how load affects the processing of distractors in interference paradigms. However, it is not clear yet how load affects the distribution of attention across space, in particular with respect to the processing of task-relevant (as opposed to task-irrelevant, in Lavie’s studies) features. Clarifying the effect of cognitive load on spatial processing, and more specifically looking into a possible difference between left and right hemispace, is the focus of the present study. Despite the absence of systematic investigations, a few studies are revealing. For example, a neuropsychological study by Bonato et al. (2010) has looked at spatial awareness under different types of load. By asking four right-hemisphere damaged patients to detect the appearance of lateralized visual targets it was shown that the number of omissions for left, contralesional targets dramatically increased when patients had to concurrently perform a second task, either visual or auditory in nature. The testing of spatial processing under such dual task conditions turned out to be a much more sensitive method to detect neglect than the classical diagnostic tests, as a striking asymmetry in visual awareness could be detected even in the absence of neglect on the classical tests (Bonato, 2015). Notably, left hemisphere damaged patients show load-induced omissions for the right hemispace (Blini et al., 2016), confirming that, after brain damage, the load effect is specific for contralesional hemispace processing.

Bonato et al. (2010) did not find any effect of increased cognitive load on spatial awareness in neurologically intact matched control participants. The clinical task may have been too easy to induce attentional asymmetries in the absence of brain damage. However, a recent review based on studies in healthy participants (Chen and Spence, 2016) suggests that, during multisensory integration, spatial processing can become asymmetric and show a rightward attentional bias under high load, in particular of perceptual origin. Peers et al. (2006) tested the effect of a secondary sound discrimination task that could either be spatial or non-spatial by nature upon a primary visual task which consisted in reporting as many as possible of six letters briefly presented in a circular arrangement. A rightward bias emerged only when adding the secondary task. Another example of spatial asymmetry under high load comes from a study looking at the crossmodal ventriloquism aftereffect (= shift in perception of spatial location; Eramudugolla et al., 2011). Besides the spatially discrepant presentation of visual and auditory stimuli which is necessary to induce the ventriloquism effect, an additional visual pattern detection task was presented. The load was manipulated by either presenting simple or complex patterns. A larger ventriloquism aftereffect was found in the high load condition, but only toward the right hemispace. A third example of spatial asymmetry under load does not involve multisensory integration, but solely focuses on the auditory domain (Golob et al., 2017). Participants had to respond to the amplitude modulation rate without paying attention to the sound location, while short term memory load was manipulated. Load presence led to clear slowing for the left compared to the right side. However, this was only true for spatial load and not for verbal load. Load can influence auditory spatial attention, but the specific pattern depends on the type of load. In a recent study (Lisi et al., 2015) a multitasking manipulation similar to the one by Bonato et al. (2010) has been applied to young, healthy participants, while they were asked to process lateralized visual targets which were masked. Under load, a trend emerged (stable across tasks yet non-significant) concerning a larger impact of the concurrent tasks upon left sided targets compared to the right. We did not find any studies looking into the effect of cognitive load on spatial attention asymmetries only within the visual modality.

Also, the load theory suggests that the nature of the imposed load is a critical determinant of how selective attention is deployed. Given that perceptual and cognitive load can have opposite effects on selective attention, it is important to use paradigms that unequivocally and exclusively manipulate one type of load. The fact that some of the tasks described above may have comprised two types of load (perceptual and cognitive) might account for some of the inconsistencies in the literature. For instance, the dual-task condition in the study of Bonato et al. (2010), in which identifying a visual or auditory stimulus was part of the secondary task, sums the effects related to dividing attention to process two different sources of information with those due to the maintenance of multiple response options, thereby increasing cognitive load. In the present study we are particularly interested in specifically isolating the effects of cognitive load, on spatial monitoring, in a context of divided attention. Yet, given the lack of paradigms designed to specifically target cognitive load, the results that are reported so far are inconclusive. One of the paradigms that allows to unequivocally manipulate cognitive load, is to preload working memory and to evaluate its impact on spatial attention during the maintenance interval in which no perceptual stimuli related to the WM task are presented.

Majerus et al. (2012) used such a paradigm in the context of an fMRI experiment. They used a letter recall task to load verbal WM and investigated how the brain responded to visual items presented during the retention interval as a function of the number of letters that had to be maintained in WM. The results showed a clear impact of WM load on the neural networks associated with spatial attention. During conditions of high WM load, the response of the temporo-parietal junction (TPJ) to the visual stimuli was suppressed. The TPJ is part of the ventral attention network and has been associated with reorienting attention toward salient or unexpected visual stimuli (Corbetta et al., 2000; Marois et al., 2000), both task-relevant and task-irrelevant (Downar et al., 2000; Corbetta and Shulman, 2002). Although this study did not systematically manipulate the location of the visual stimuli and although no behavioral measurements were obtained, these results are suggestive of a potentially influential role of cognitive load on spatial attention. Based on the fact that damage to the right TPJ is considered to be the crucial reason for the rightward attentional bias in neglect, it can *a priori* be predicted that the right-lateralized reduction of TPJ activity induced by high cognitive load would lead to a worse detection of stimuli in the left hemispace compared to those in the right hemispace. The current study was designed to test this prediction. Specifically, we investigated spatial processing differences for left-sided versus right-sided stimuli when verbal WM is loaded with more (high load) or less (low load) items. Although spatial selective attention has been widely investigated while manipulating the load, to our knowledge, no study has looked into the effect of WM load on spatial attention concerning left–right asymmetries. Specifically, we used an approach in which a detection task was used to measure spatial attention in the context of a WM letter recall task that was used to preload verbal WM. Moreover, we aimed at correlating the size of the space-load interaction with the impact load had at the individual level.

## Experiment 1

### Materials and Methods

#### Participants and Apparatus

Twenty participants (all university students, six males, *M* = 23.45 years, *SD* = 3.59) gave informed consent and were paid €10 to participate. None of the participants were aware of the purpose of the experiment. Two participants were left-handed (based on self-report) and all had normal or corrected-to-normal vision. We conducted the experiment on a Dell laptop running E-prime 2.0.8.90 software (Psychology Software Tools, Inc.^1^). The stimuli were presented on an external monitor (19-inch wide-screen LCD, Dell) and responses were given on an external keyboard. The distance from the participant to the screen was approximately 60 cm. All stimuli were presented in black on a white background.

#### Procedure

**FIGURE 1:**
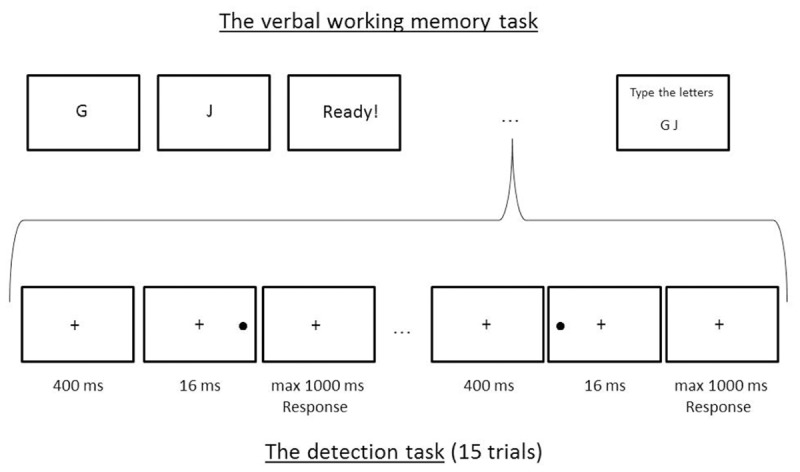
An overview of a low WM load (two letters) trial in the WM task, which consists of 15 detection trials. The images are not in scale.

The experiment consisted of a detection task that was presented during the maintenance interval of a parallel WM task (see **Figure 1**).

##### Working memory task

Before the start of the detection task, a sequence of two (low WM load) or six (high WM load) letters had to be memorized. The letters were presented one at a time, in the middle of the screen. None of the letters were vowels. No letters were repeated within a sequence. Participants were instructed to remember the letters in exactly the same order. They could go through the letters at a self-determined pace. Once all the letters had been seen, the detection task (15 trials) started. After finishing the detection task, participants had to recall the sequence of letters and type it using a standard keyboard. Their response appeared on the screen and if necessary, corrections could be made. There were 40 WM sets, half of which were low WM load trials and half of which were high WM load trials. It was a block design, alternating the load condition, with five sets per block and eight blocks in total. Participants were always informed in advance about the difficulty level of the block (easy or hard). Half of the participants started with an easy block and half of them with a hard block. Between each block participants could take a short break if necessary.

##### Detection task

The detection task was performed during the maintenance interval of the WM task and always comprised 15 trials. Thus, after the participants went through the letters they had to remember for the WM task, the detection task began. During the whole detection task, a fixation cross (height 14 mm, 1.33° of visual angle) was present in the center of the screen. Every trial started with the text “Klaar!” (“Ready!”) displayed for 1500 ms on top of the fixation cross. A target stimulus appeared 400 ms after the text disappeared. The target stimulus (black dot) had a diameter of 9 mm (0.86°), and was presented for 16 ms (synchronized with the 60 Hz refresh rate of the screen) either on the left or the right side of the screen. In one third of trials, no target was presented. Participants were instructed to press the spacebar as soon as they saw the target, irrespective of its position. They maximally had 1000 ms to give a response and as soon as a response was given, the next detection trial started. They were explicitly asked to perform this detection task with the index finger of their dominant hand. For every detection task during a WM trial, the position of the target was balanced and randomized across the 15 detection trials. The experiment encompassed 600 target detection trials: 200 with a left-sided target, 200 right-sided, and 200 without any target. Half the trials were performed while under low WM load and half under high WM load.

#### Data Analysis

We used R (R Core Team, 2016) and lme4 (Bates et al., 2015) to perform generalized linear mixed effects (GLME) analyses. In case the dependent variable was dichotomous (accuracy), we used logistic regression analyses. Both for the fixed and random effects, the chi-square statistics and the corresponding *p*-values were acquired by the likelihood ratio test. The full model was compared with the model without the effect at test. All the results were controlled for age, gender, and handedness.

### Results

First, to investigate the performance on the WM task, we entered the accuracies into a GLME model with a random intercept across participants and WM load as a fixed effects predictor. There was a significant main effect of Load, χ^2^(1, *N* = 20) = 15.87, *p* < 0.001. Accuracy in recalling the WM sequence was higher in the low WM load condition (*M* = 96.5%, *SD* = 4.9%) compared to the high WM load condition (*M* = 84.3%, *SD* = 12.8%), indicating that the load manipulation was successfully implemented. Secondly, participants performed well on the detection task with an average of 97% correctly detected targets. The error-rate (omissions and false alarms) was similar for the low load (2.9%) and for the high load (3.1%) condition.

**FIGURE 2:**
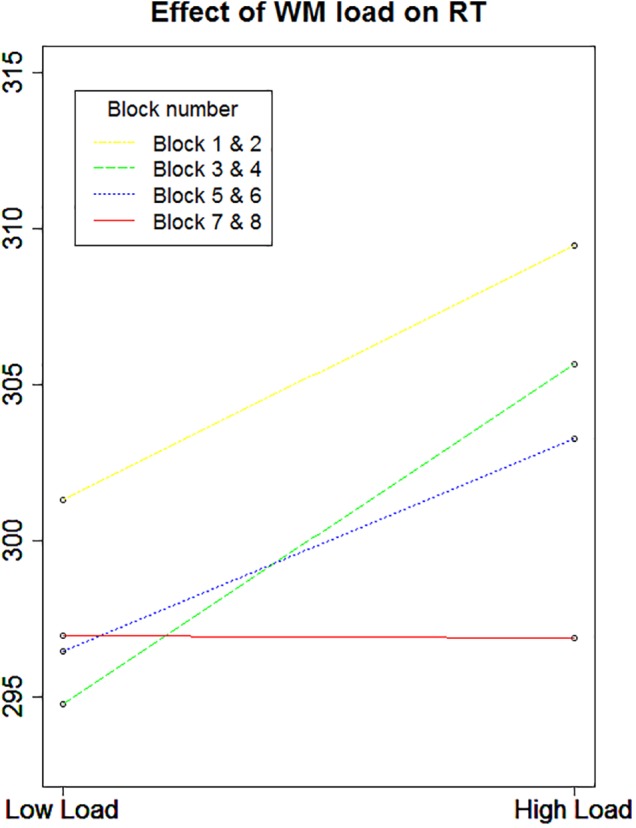
Average RTs as a function of block and load (Experiment 1). A significant interaction between WM load and block (*p* = 0.012) emerged. Since the load conditions alternated, we chose to look at the blocks in pairs and as such a low and a high WM load block are always coupled. Results show that the effect of WM load on RT decreases over time and even completely disappears in the last two blocks. As a consequence, it seems difficult to assume that the load manipulation was still effective at that time point.

**FIGURE 3:**
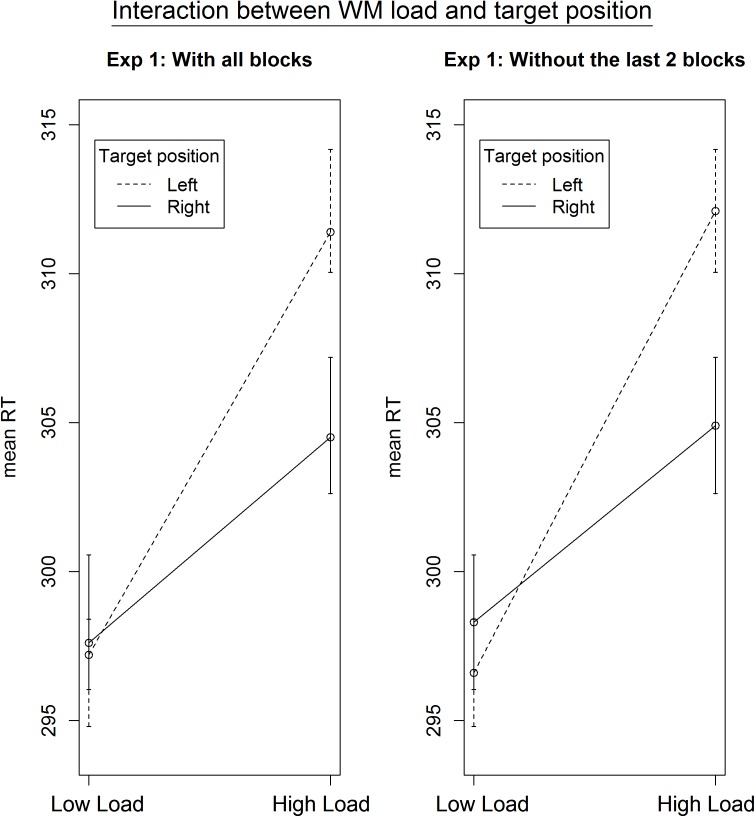
Average RTs as a function of target position and load (Experiment 1). The left panel represents all blocks, the one on the right only those where we could ensure that the WM manipulation was effective. The interaction between WM load and target position only becomes significant (from *p* = 0.07 to *p* = 0.049) when excluding the last two blocks from analysis. The effect of load is larger for stimuli presented in the left vs the right hemispace.

**FIGURE 4:**
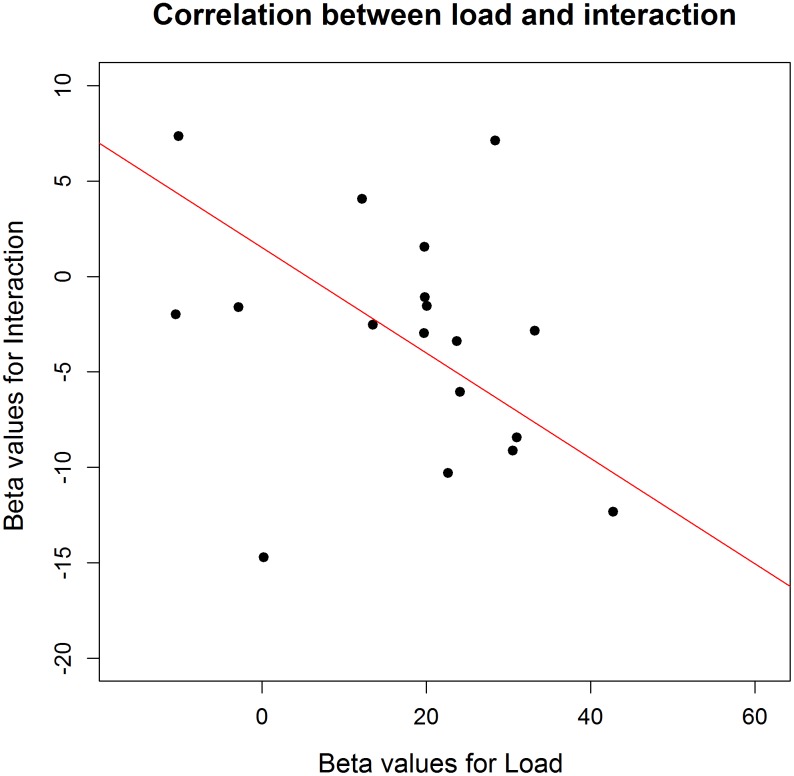
Correlation between the effect sizes of load and those of the interaction between position and load on an individual level (Experiment 1). This correlation shows that the interaction was stronger in those participants who presented a stronger effect of increased load.

To investigate the influence of the WM load manipulation on the detection task, the RTs on the detection task were entered in a GLME model, with a random intercept per participant, a random slope for Load and for Position (left versus right) and, as fixed effect predictors, Block and the interaction between Load and Position. Error trials (3.06% of the data) and trials with RTs below 100 ms (0.6% of the data) were excluded from further analysis. Additionally, the trials in which no target appeared were left out from further analysis. Finally, the detection trials during an incorrect WM trial were also omitted from further analysis, due to the impossibility to determine whether a WM load was present during those detection trials. There was a significant main effect of Load, χ^2^(1, *N* = 20) = 4.47, *p* = 0.034. RTs were slower under high WM load (*M* = 307.7 ms, *SD* = 88.3) compared to low WM load (*M* = 297.3 ms, *SD* = 79.1), which again confirms that the WM load manipulation was successful and that its effect was present during the detection task. We found no main effect of Position, χ^2^(1, *N* = 20) = 0.69, *p* = 0.41. Next, there was a significant main effect of Block, χ^2^(1, *N* = 20) = 14.73, *p* < 0.001. Participants became faster toward the end of the experiment, indicating a learning effect. When looking at the data in more detail, we found a significant interaction between Load and Block, χ^2^(1, *N* = 20) = 6.28, *p* = 0.012, with the effect of load disappearing in the last blocks. We reasoned that this might explain why the interaction between Load and Position was not significant, χ^2^(1, *N* = 20) = 3.14, *p* = 0.07. As illustrated in **Figure 2**, the main effect of Load disappeared during the last two blocks, in which participants reacted equally fast on targets during low WM load trials as during high WM load trials. As we cannot be sure that the load manipulation was still effective throughout those last two blocks, we repeated the same analyses without the last two blocks. All analyses revealed very similar results, and the interaction between Load and Position turned out to be significant, χ^2^(1, *N* = 20) = 3.86, *p* = 0.049. The slope for the main effect of Load was steeper for targets appearing on the left than on the right (**Figure 3**). It shows that the difference in processing stimuli under low versus high load is larger for stimuli in the left hemifield compared to stimuli in the right hemifield. If this interaction is indeed a consequence of a high WM load, we expect to find a correlation across subjects between the size of the load and the size of the interaction between load and position. Therefore, we correlated the beta values (from the GLME model) of load with those of the interaction for each participant. The results show that participants who experienced a bigger impact of load, also show a stronger interaction, *r*(18) = -0.64, *p* = 0.002 (**Figure 4**).

### Discussion

Experiment 1 investigated whether cognitive load influences spatial processing differently for stimuli in the left or the right hemispace. We found that adding WM load had a greater effect on detection speed for a target presented on the left compared to the right side of a monitor. The interaction between load and position was significant as long as an effect of load was present. The disappearance of the load effect in the last two blocks might be due to learning, although we would have expected a gradual difference across blocks instead of a sudden drop in reaction times.

This pattern of results is suggestive of an asymmetrical attentional bias with a relative disadvantage for the left vs the right hemispace as a result of a high cognitive load. These results could in principle be explained by hand compatibility, as left hemisphere lateralized motor activity could imply an increased sensitivity for the right hemispace. Participants were instructed to respond with their dominant hand, which was mostly the right hand (except for two left-handed participants). Thus, a facilitation effect could be present for right-sided targets due to responses being performed with the right hand rather than to effector-independent visuospatial asymmetries. To explore this alternative explanation, we conducted a second, almost identical, experiment. The only difference consisted in the use of the left (non-dominant) hand for responding. Finding a larger impact of load for targets on the right would be in favor of the response compatibility hypothesis. On the other hand, in case we would again find a larger impact of load on left-sided targets, it would support our original hypothesis about asymmetrical spatial processing under cognitive load.

## Experiment 2

### Materials and Methods

Twenty participants (all university students, three males, *M* = 24.25 years, *SD* = 4.10) gave informed consent and were paid €10 to participate. None of the participants were aware of the purpose of the experiment. All participants were right-handed (based on self-report) and all had normal or corrected-to-normal vision. The method was identical to that of Experiment 1, with the exception that participants had to respond with their non-dominant hand during the detection task. The data analyses and model building were also identical.

### Results

To investigate the performance on the WM task, we entered the accuracies into a GLME model with a random intercept across participants and WM load as a fixed effects predictor. There was a significant main effect of Load, χ^2^(1, *N* = 20) = 21.4, *p* < 0.001. Accuracy in recalling the WM sequence was higher in the low WM load condition (*M* = 99%, *SD* = 3.5%) compared to the high WM load condition (*M* = 82.75%, *SD* = 20.4%), indicating that the load manipulation was successfully implemented.

**FIGURE 5:**
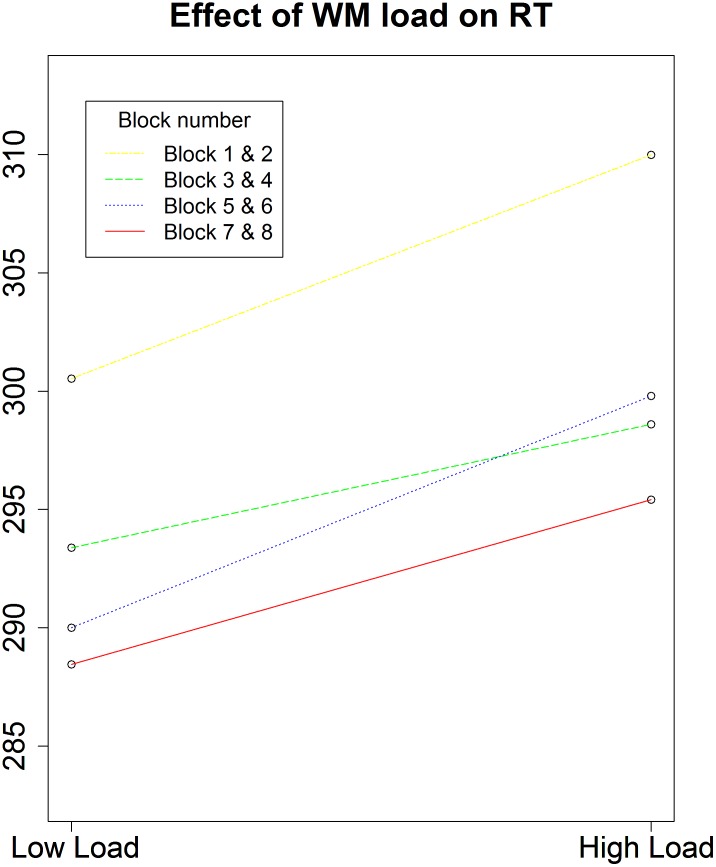
Average RTs as a function of block and load (Experiment 2). No significant interaction between WM load and block (*p* = 0.196) emerged. Results show that the effect of WM load on RT remains present across the whole experiment.

**FIGURE 6:**
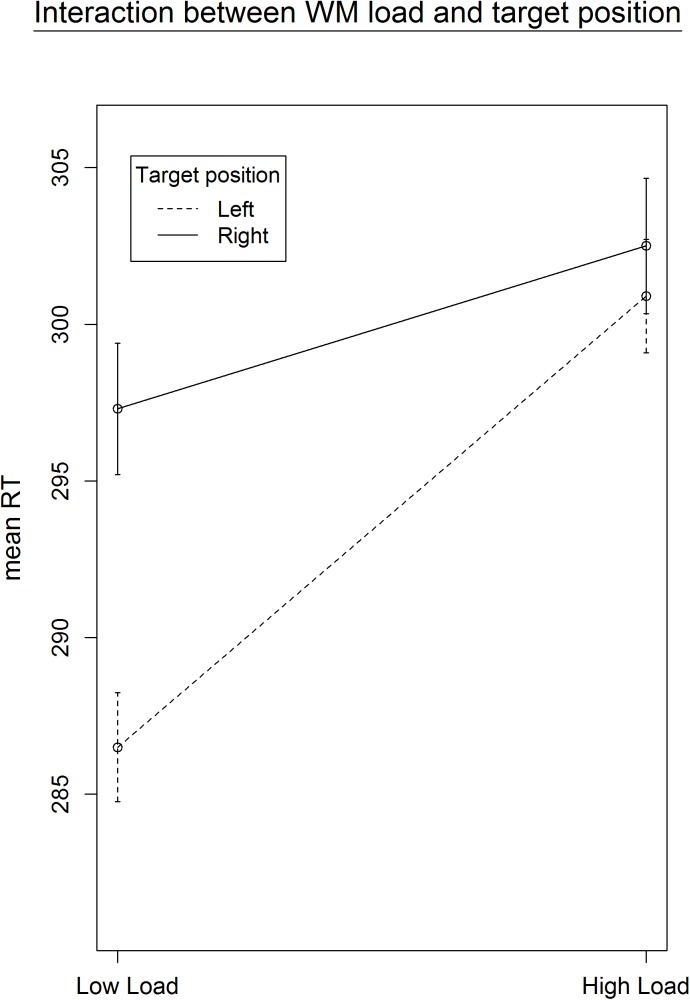
Average RTs for target position and load (Experiment 2). As in Experiment 1, (dominant hand response), we also found in Experiment 2 (non-dominant hand response) a significant interaction between WM load and target position (*p* = 0.001). Again, load affected the processing of stimuli presented in the left hemispace more than those in the right hemispace.

**FIGURE 7:**
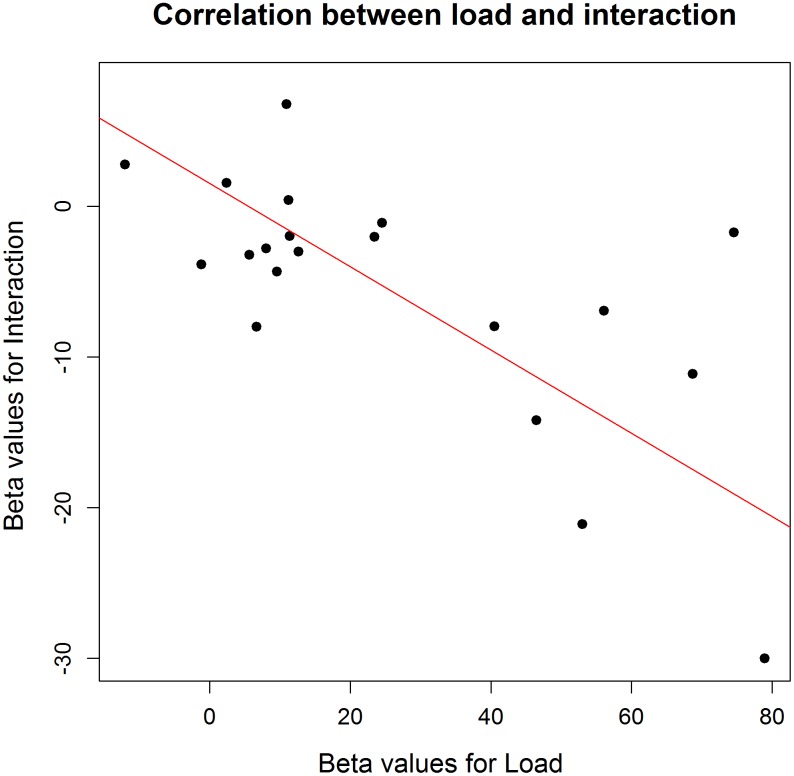
Correlation between the effect sizes of load and those of the interaction between position and load at individual level (Experiment 2). The interaction was stronger in those participants who encountered a larger effect of increased load. It also shows that the interaction was reliably present across participants.

Participants correctly detected 98.6% of targets. The error-rate was similar for the low load (1.3%) and for the high load (1.6%) condition. Together with error trials, trials with RTs below 100 ms (1.7% of the data) were excluded from further analysis. As for Experiment 1, the no-target trials and the detection trials during an incorrect WM trial were omitted from further analysis. To investigate the influence of the WM load manipulation on the detection task, the RTs on the detection task were entered in a GLME model, with a random intercept per participant, a random slope for Load and for Position and as fixed effect predictors Block and the interaction between Load and Position. A significant main effect of Load emerged, χ^2^(1, *N* = 20) = 7.96, *p* = 0.005. RTs were slower under high WM load (*M* = 301.7 ms, *SD* = 87.4) compared to low WM load (*M* = 291.4 ms, *SD* = 71.4), which again confirms that the WM load manipulation was successful and that its effect was present during the detection task. Position showed a trend toward a response compatibility-like effect with faster responses to left-sided targets, χ^2^(1, *N* = 20) = 3.26, *p* = 0.07. Next, there was a significant main effect of Block, χ^2^(1, *N* = 20) = 71.37, *p* < 0.001 (**Figure 5**). Participants became faster toward the end of the experiment, indicating a learning effect. There was no significant interaction between Load and Block, χ^2^(1, *N* = 20) = 1.67, *p* = 0.196. There was a significant interaction between Load and Position, χ^2^(1, *N* = 20) = 10.25, *p* = 0.001. As illustrated in **Figure 6**, Load has a bigger impact on the reaction times of targets appearing on the left than on the right. We also looked at the correlation between the size of the load and the size of the interaction at individual level. We correlated the individual beta values (from the GLME model) of load with those of the interaction between load and position. The correlation was significant, *r*(18) = -0.68, *p* < 0.001. We then checked for the presence of outliers. Only one participant could be considered an outlier (>2.5 *SD*). Its exclusion did not considerably change the results, *r*(17) = -0.58, *p* = 0.005 (**Figure 7**). As an extra analysis, we combined the data of both experiments. Our aim was to investigate the effect of between-subject variable Hand Response (Experiment 1 and Experiment 2) on the interaction between Load and Position. To do so, we entered the RTs of the two detection tasks into a GLME model with a random intercept across participants and a three-way interaction between Load, Position, and Hand Response as fixed effects predictors. While the interaction between Load and Position remains significant, χ^2^(1, *N* = 40) = 14.01, *p* < 0.001 there was no hint of a significant three-way interaction, χ^2^(1, *N* = 40) = 0.10, *p* > 0.05.

### Discussion

In Experiment 2, participants responded using their non-dominant hand (left). We again found that cognitive load had a greater effect on the stimuli presented on the left versus the right side. When comparing **Figures 3**, **5**, one may observe that in the first experiment, participants were faster at the ipsilateral location only in the high load condition while in the second experiment this finding was present for the low load condition. To evaluate the effect of hand response on these effects, we analyzed the data of the two experiments combined. The interaction between the position of the target and the WM load we found was not influenced by the hand performing the response. It is therefore possible to conclude that the difference present across experiments is purely an additive effect. A tentative explanation for this additive effect could be based on hemispheric activation. The biggest advantage emerges in the low WM load condition where targets appear left and participants have to respond with their left hand. Every aspect of this condition is primarily processed by the right hemisphere which is dominant for some aspects of spatial attention (Kinsbourne, 1970a). This hemisphere-driven effect could lead to the observed faster reaction times. The conceptual replication of the findings of Experiment 1 in Experiment 2 provides additional support for hypothesis of an attentional origin for asymmetric spatial processing under cognitive load.

## General Discussion

The aim of this study was to investigate how spatial attention would be affected by different levels of cognitive load. We hypothesized that the processing of information presented within the left hemispace would suffer more from high load compared to information within the right hemispace. We tested this hypothesis by manipulating the verbal WM load via a letter recall task to then measure spatial attention effectiveness via a detection task. Participants first had to memorize a sequence of letters and while their WM was loaded, they performed a detection task in which they had to detect briefly presented targets appearing either on the left or on the right side. In the first experiment, we found that high WM load slowed down the detection of left-sided targets more than the detection of right-sided ones, which corresponded with our hypothesis. To exclude an alternative explanation of hand response, we conducted a second experiment with a different response mapping. While in the first experiment participants had to respond with their dominant hand, we asked the participants of the second experiment to respond with their non-dominant hand. Also in the second experiment, results showed a significant interaction between the WM load condition and the target position with a more evident effect for processing information in the left hemispace. We analyzed the data of the two experiments together and found no influence of hand response on the described interaction between the position of the target and the amount of WM load present. In the two experiments, our hypothesis is further corroborated by the fact that the individual strength of the load effect correlated with the individual degree of asymmetry in spatial processing.

We point out that there are individual differences we cannot control for and which might affect performance. For example, someone’s anxiety level can influence the effect of perceptual load (Moriya and Tanno, 2010; Sadeh and Bredemeier, 2011) and it can be affected by many more both inter- and intra-individual differences (Shipstead et al., 2012; Marciano and Yeshurun, 2017). An advantage of this study compared to some other load studies (e.g., Lavie, 1995) is that we have an independent measurement of the load manipulation and can correlate it at the individual level to the spatial processing effect we are interested in. In the current study we were interested in the effect of cognitive load on the symmetry of spatial processing. We manipulated the load level by a verbal WM task in which either two or six letters had to be remembered. While memorizing six letters can generally be stated as more difficult and thus inducing a high load, there is quite some variation between individuals when it comes to determining how difficult the high load condition is. This inter-individual difference is reflected both in the accuracy score on the WM task itself as in the reaction times during the detection task. To be sure that the asymmetrical spatial processing we observed could be attributed to the presence of high cognitive load, we expected that, at the individual level, the size of the load effect would have been related to the size of the asymmetry. That was exactly what we found when we correlated the effect of load on reaction time in the detection task with the interaction between load and position.

Majerus et al. (2012), showed TPJ deactivation under conditions of high cognitive load. The asymmetrical spatial processing we described is compatible with the possibility of right TPJ suppression under load (Shulman et al., 2010). We can thus state that our behavioral results are in accordance with existing fMRI evidence of an interaction between spatial attention and WM plausibly occurring in the TPJ (Anticevic et al., 2010). From a model perspective, to explain the mismatch between neural and functional impairment in neglect patients, Corbetta and Shulman (2011) suggest a modulation of the ventral attention network (VAN) on the dorsal attention network (DAN). We propose that this modulation of the VAN on the DAN could be mediated by cognitive load. Keeping in mind that the TPJ, part of the VAN, is suggested to be dominant in the right hemisphere, load-dependent de-activation in the (right-lateralized) VAN should translate – at the behavioral level – to worse spatial processing in the left hemispace compared to the right hemispace. This is compatible with the prominent disturbances in contralesional spatial processing right hemisphere damaged patients show. This also reflects the results found in this study with healthy participants where the load manipulation was clearly WM oriented. To gain more insight into the underlying neural mechanisms responsible for the current findings, it would be interesting to perform an fMRI study with a similar behavioral design.

Our results are also in line with studies looking at the effect of perceptual load on multisensory spatial processing (Chen and Spence, 2016), although in those studies it was not possible to disentangle the perceptual and cognitive load from each other. This makes it difficult to attribute the asymmetric effect to one specific type of load. In our experiment we deliberately chose to use a verbal WM load to avoid any perceptual load influence. After having ensured the cognitive nature of the load manipulation, the question becomes whether the current findings are specifically related to the verbal nature of the WM load. Memorizing a sequence of letters has a verbal nature and will consequently activate our left hemisphere more because of its dominance for language. Spreading of activation within the left hemisphere could offer an alternative explanation for why left-sided targets are processed slower compared to right-sided ones (Tokimura et al., 1996; Seyal et al., 1999; Meister et al., 2003). This reasoning expands Kinsbourne’s (1970a,b) findings concerning significant asymmetries in the performance on a visual task in favor of the right side in the presence of verbal load as opposed to no load. However, one could argue that both load conditions are of a verbal nature, and the corresponding verbal components should not differentially interact with the spatial component and thus it should not confound the results. Of course one could still argue that a high WM condition is more demanding and might activate language more than the low load condition. This question remains open for future investigation.

Resulting from our underlying neural hypothesis, we might prefer to frame our findings as due to a disadvantage for the left hemispace rather than a rightward facilitation. One way to empirically differentiate between an advantage for the right versus a disadvantage for the left hemispace would be to add central targets and use this as a reference point to compare the reaction times of the left and right targets with.

## Conclusion

We investigated the effect of cognitive load on spatial processing in the left versus the right hemispace. We observed a different impact of verbal WM load on spatial processing. Load affected more the left than the right hemispace. A correlation between the load effect and the interaction on the individual level further supported our hypothesis that there is an attentional origin for the asymmetric spatial processing we observed. Further research might allow to test whether the effect is specific for the verbal nature of the WM load.

## Ethics Statement

This study was carried out in accordance with the recommendations of the Ethical Committee of the Faculty of Psychology and Educational Sciences of UGhent with written informed consent from all subjects in accordance with the Declaration of Helsinki. The protocol was approved by the Ethical Committee of the Faculty of Psychology and Educational Sciences of UGhent.

## Author Contributions

LN and WF developed the study concept. LN programmed the experiment, collected the data, and performed the data analysis. LN, WF, MB drafted the manuscript. WF and MB provided critical feedback on different versions of the manuscript. All authors listed have made substantial, direct and intellectual contribution to the work, and approved it for publication.

## Conflict of Interest Statement

The authors declare that the research was conducted in the absence of any commercial or financial relationships that could be construed as a potential conflict of interest.

## Footnotes

1 www.pstnet.com
